# Supplementary material for: Plasmid fitness costs are caused by specific genetic conflicts enabling resolution by compensatory mutation
Source: PLoS Biol. 2021 Oct 13;19(10):e3001225. doi: 10.1371/journal.pbio.3001225 (PMC8544851; doi:10.1371/journal.pbio.3001225)
Supplement: S1 Text — (PDF) [file pbio.3001225.s009.pdf]

S1 Text

Supplementary Results and Discussion for: Plasmid fitness costs are caused by specific genetic conflicts enabling resolution by compensatory mutation

James P. J. Hall, Rosanna C. T. Wright, Ellie Harrison, Katie J. Muddiman, A. Jamie Wood, Steve Paterson, Michael A. Brockhurst

Compensatory mutations detected in previous studies

Hall et al. 2017; Hall et al. 2018 (pQBR57)

Previously, we evolved *Pseudomonas fluorescens* SBW25 and *P. putida* KT2440 in soil microcosms for ~440 generations with and without the mercury resistance conjugative plasmid pQBR57 (Hall et al. 2016). Whole-genome sequencing of evolved clones identified two loci that were frequently mutated in independent populations and were associated with pQBR57 carriage (Hall et al. 2018). Specifically, mutations to the plasmid gene *PQBR57_0059* appeared in 9/36 (25%) of populations where the plasmid was initially present, with mutations present in both *P. fluorescens* and *P. putida*. Disruptions to *PQBR57_0059* occurred both as single nucleotide mutations and as transposon insertions, and targeted both the open reading frame (ORF) and the region 5' of the ORF between the predicted promoter (Solovyev & Salamov, 2011) and the start codon. The other locus was the chromosomal gene *PFLU4242*, present in *P. fluorescens* SBW25 and with no homologue in *P. putida* KT2440, which was targeted in 11/34 (32%) of populations, with single nucleotide mutations including frameshifts occurring across the ORF. Neither locus was mutated in plasmid-free treatments, and no other mutations were associated with plasmid carriage in this experiment. Interestingly, populations evolved in the absence of mercury were more likely to have plasmid-borne compensations than chromosomal compensations (4/6 compensated clones; 66%), compared with populations evolved under

positive (mercury) selection (3/12 compensated clones; 25%), though we lacked sufficient replication to test this rigorously.

Hall et al. 2019 (pQBR55)

Hall et al. (2019) found mutations in *P. fluorescens* SBW25 GacA/S system, in *PFLU4242*, or in RNA polymerase, in independent clones ameliorating the heavy fitness cost of pQBR55. Mutations to PFLU4242 were most common (10/14 ameliorated clones). Mutations to RNA polymerase were not as effective in ameliorating pQBR55 as the *gacA/S* or *PFLU4242* mutations, and may have emerged in a minority of clones (2/14 ameliorated clones) due to the extreme population bottlenecks employed in this study.

Harrison et al. 2015 (pQBR103)

Harrison et al. (2015) found that the main signature of *P. fluorescens* SBW25 compensation of pQBR103 was mutation to the *gacA/S* two-component regulator. However, clones from a quarter of the populations that did not have a *gacA/S* mutation (2/36 of the total populations) acquired instead a mutation in *PFLU4242*.

Harrison et al. 2017 (pQBR103, with phage phi-2)

Harrison et al. (2017) found *PFLU4242* mutations in half of the populations evolved with pQBR103 that did not have a mutation in *gacA/S* (3/12 of total populations). *PFLU4242* mutations were more common than *gacA/S* mutations in the phage-evolved populations (2/3; 66%) than in the no-phage populations (1/6; 17%), but there was not sufficient replication to test these distributions statistically.

Carrilero et al. 2021 (pQBR103, pQBR57, or both pQBR103 and pQBR57)

Carrilero et al. (2021) found *gacA*, *gacS*, and *PFLU4242* mutations occurring in all evolved populations, regardless of whether they evolved with pQBR57, pQBR103, or both pQBR57 and pQBR103. There was no evidence for differential compensatory evolution according to treatment.

The list of compiled mutations presented in Figure 1 is available as part of the dataset for this study (**https://github.com/jpjh/COMPMUT/** ; doi: **10.17638/datacat.liverpool.ac.uk/1275**)

Diverse, naturally-occurring PQBR57_0059 loss-of-function mutants also enhance plasmid maintenance

Naturally-emerging pQBR57 variants from the evolution experiment, reported in Hall et al. (2017, 2018) were selected on the basis of either having an intact PQBR57_0059 gene (5 variants), or having a mutation in that gene or immediately upstream (7 variants). Most of these pQBR57 variants also contained second-site mutations or transposon insertions. Plasmids were conjugated into an ancestral strain in triplicate and cultured in soil microcosms alongside a differently-*lacZ*-labelled plasmid-free competitor for 5 transfers following the protocol in Hall et al. (2017). Counts of (initially) plasmid-bearing and (initially) plasmid-free strains was achieved by spreading samples on plates containing X-gal at each transfer, and plasmid carriage in both subpopulations was assessed by replica-plating onto plates containing mercury (**Fig S2**). These experiments were run alongside similar experiments performed with the ancestral pQBR57 variant (Hall et al. 2017). We fitted a linear mixed-effects model to cumulative plasmid frequency, with PQBR57_0059 status as a fixed effect, and variant as a random effect. Disrupted PQBR57_0059 variants had a significantly higher cumulative plasmid frequency (LMM, effect of PQBR57_0059 disruption χ^2 = 5.6, p = 0.02), though there was substantial variation between variants, with variant C01, which had gained a Tn6291 insertion, as an outlier.

The overall pattern held even if the ancestral variant was not included in the analysis (χ^2 = 3.95, p = 0.047).

Supplementary References

Carrilero, L., Kottara, A., Guymer, D., Harrison, E., Hall, J. P. J., & Brockhurst, M. A. (2021). Positive Selection Inhibits Plasmid Coexistence in Bacterial Genomes. *mBio*, *12*(3). https://doi.org/10.1128/mBio.00558-21

Hall, J. P. J., Williams, D., Paterson, S., Harrison, E., & Brockhurst, M. A. (2017). Positive selection inhibits gene mobilisation and transfer in soil bacterial communities. *Nature Ecology & Evolution*, *1*(9), 1348–1353. https://doi.org/10.1038/s41559-017-0250-3

Hall, J. P. J., Harrison, E., & Brockhurst, M. A. (2018). Competitive species interactions constrain abiotic adaptation in a bacterial soil community. *Evolution Letters*, *2*(6), 580–589. https://doi.org/10.1002/evl3.83

Hall, J. P. J., Wright, R. C. T., Guymer, D., Harrison, E., & Brockhurst, M. A. (2019). Extremely fast amelioration of plasmid fitness costs by multiple functionally diverse pathways. *Microbiology*. https://doi.org/10.1099/mic.0.000862

Harrison, E., Hall, J. P. J., Paterson, S., Spiers, A. J., & Brockhurst, M. A. (2017). Conflicting selection alters the trajectory of molecular evolution in a tripartite bacteria-plasmid-phage interaction. *Molecular Ecology*, *26*(10), 2757–2764. https://doi.org/10.1111/mec.14080

Harrison, E., Guymer, D., Spiers, A. J., Paterson, S., & Brockhurst, M. A. (2015). Parallel compensatory evolution stabilizes plasmids across the parasitism-mutualism continuum. *Current Biology: CB*, *25*(15), 2034–2039. https://doi.org/10.1016/j.cub.2015.06.024

Solovyev, V., & Salamov, A. (2011). AUTOMATIC ANNOTATION OF MICROBIAL GENOMES AND METAGENOMIC SEQUENCES. In R. W. Li (Ed.), *In Metagenomics and its Applications in*

Agriculture, Biomedicine and Environmental Studies (pp. 61–78). Nova Science Publishers. https://www.researchgate.net/publication/259450599
