# Supplementary material for: Plasmid fitness costs are caused by specific genetic conflicts enabling resolution by compensatory mutation
Source: PLoS Biol. 2021 Oct 13;19(10):e3001225. doi: 10.1371/journal.pbio.3001225 (PMC8544851; doi:10.1371/journal.pbio.3001225)
Supplement: Attachment — Submitted filename: COMPMUT_manuscript_REVISIONTABLE_SUBMITTED.pdf [file pbio.3001225.s010.pdf]

We thank all Reviewers for their interest in the work, and their helpful and constructive comments. We believe that the changes we have made in response to their suggestions have greatly improved the manuscript. We hope that we have addressed each of their concerns in the point-by-point table below. In addition, images and captions have been reformatted as requested by the Publications Assistant.

Reviewer 1

**Suggestion**	**Response**
My only "relevant" criticism to this manuscript is that it may result a bit too long/dense, and while a plasmid biologists like myself has REALLY enjoyed reading it, a more "general" reader may have a little trouble navigating the paper. The authors could maybe try to streamline the manuscript (results and discussion) and maybe move some parts to the supplementary material (for example "Plasmid compensatory evolution need not negatively affect plasmid transmission"?).	This manuscript is the culmination of more than a decade of our work on this plasmid-host association. We have opted to publish this as a single paper in a journal without arbitrary page/word-limits so that we could present as complete an investigation as possible. As such, while we accept that the paper is long, we feel that each piece of presented data adds an important piece to the overall picture. We would therefore prefer to retain the current structure, but will of course follow editorial guidance if it is decided that shortening the paper is required for publication.
Line 39, helping to explain?	Thanks — we've taken the Reviewer's suggestion.
Line 265 "of plasmid compensation"?	Thanks for spotting this, now corrected.
Line 374, close parenthesis?	Thanks for spotting this, now corrected.
Line 486, Figure 5 instead of Figure 4, which also affects the numbering of the following figures.	Thanks — fixed now.
Figure 4A, full names in legend may help the reader.	We assume the reviewer means what is now Figure 5 (now we have sorted the figure numbering out). The full names for the regions are now provided.
The interaction between pQBR57_0059 and the upstream region of the parAb genes seems likely, but it would be great to confirm if with an electrophoretic mobility shift assay. I understand however that this may be beyond of the scope of this work.	We agree that this would be an interesting subject of future investigations. However, due to the scale of the additional experimental work involved it is unfortunately beyond the scope of the current study.
If tailocin PFLU1169 is a crucial source of plasmid costs one may expect mutations inactivating this particular gene in compensated clones. Have the authors ever found mutations there?	No, we have not observed mutations in PFLU1169. It is likely that the SOS response has various deleterious effects, of which activating expression of PFLU1169 is just one, albeit an important and very costly example, as our data show. We believe that this explains why mutations in PFLU1169 have not been found in our experimental evolution studies, since mutations that prevent the SOS response from becoming triggered (i.e. to gacA/S or PFLU4242) are more successful. We now include a couple of words to this effect in the discussion (lines 722ff.)
Figure 8 is important, because it summarises the numerous findings of the study, and of previous studies, in a single model, but some of the connections are a bit difficult to understand for me. For example: "(B) PFLU4242 activates the SOS response, probably through causing DNA breaks". The authors showed that inactivation of PFLU4242 produced the silencing of the plasmid-induced SOS response, but that does not necessarily indicate that PFLU4242 activates the SOS response. In fact the expression level of PFLU4242 does not increase in the presence of the plasmids (Fig S5). Its true that the authors speculate about the potential role of PFLU4242 an anti-phage nuclease, but I think that what has been shown for sure is that plasmids activate the SOS response, and that compensatory mutations silence it back. Therefore I think the arrows form the plasmids should maybe point directly the SOS response instead of to the GacAS and PFLU4242 (and these should be linked with the silencing of the SOS response), but this is just a suggestion.	Though the plasmids do activate the SOS response, this depends on a functional PFLU4242 gene also being present in the genome. Loss-of-function mutation of PFLU4242 silences the SOS response (Figure 3). We believe that this is congruent with saying that wild-type PFLU4242 activates the SOS response, though we also accept the Reviewer's point that this process is really an interaction between functional PFLU4242 and (either) plasmid. We have therefore modified Figure 9 (previously mislabeled Figure 8) so that the arrow indicating activation of the SOS response now comes from the arrows indicating conflict between plasmids and PFLU4242. We also now make clear that this model is a proposal, since it includes some speculative interactions (title to Figure 9).

Reviewer 2

<table>
<tr><th>Suggestion</th><th>Response</th></tr>
<tr><td>It is not very satisfying that higher expression of par genes on a plasmid would decrease the fitness cost of that plasmid, and not rather decrease the loss rate (see also comment 2: were the strains plated on medium with plasmid, or was the loss rate really unchanged?). The authors write in Fig. 7" Mutation of PQBR57_0059 increases expression of par, which reduces the disruption of pQBR57". In the discussion the authors suggest possible functions of these genes other than plasmid partitioning. Are the authors sure that the competition assays were not confounded by plasmid loss during the assays? And can they say a bit more about how similar the genes are to known partitioning genes and what the likelihood is that they are involved in partitioning, or rather may have a different function as speculated?</td><td>

We are confident that mutations to PQBR57_0059 (and thus upregulation of *par*) do not affect segregational loss, at least not to a degree that could explain our results. To test for the impact of segregation, we regularly replica plated endpoint competition plates onto selective media and found that segregants were very rare. For example, in Figure S2 we performed serial transfer of different pQBR57 variants over the course of approximately one month, and found no evidence of significant segregation, as assessed by regular replica plating onto selective media, and endpoint PCR tests for plasmid presence. These patterns were also consistent with our previous studies (e.g. Hall et al. 2016).

However, as this is a subject that has come up several times in presentations of this work, we decided to address it directly with a new experiment, wherein we performed serial transfer of strains ectopically expressing pQBR57 par and assessed carriage of pQBR57 or pQBR103 by replica plating over four growth cycles. While we did see some pQBR103 segregants emerge, consistent with our previous work (e.g. Hall et al. 2015, Harrison et al. 2015), we found no significant effect of ectopic pQBR57 *par* expression on the emergence of segregants, indicating that it is more likely that *par* works directly to ameliorate fitness costs, rather that these effects emerging indirectly as a consequence of plasmid loss. See Figure S8 and lines 666ff.

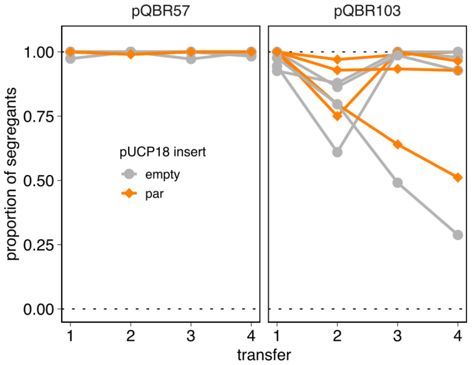

Fig S8. Overexpression of pQBR57 par genes does not significantly affect plasmid loss. *Single colonies derived from independent transformations/conjugations were established in KB media supplemented with 50 µg/ml kanamycin (to select for maintenance of the pUCP18 expression vector), and transferred 1:100 into fresh media every 2 days for four transfers (approx. 25 generations). At each transfer, a sample was spread on KB agar, and after colonies had grown, was replica-plated onto KB supplemented with 100 µM HgCl2. Plots show proportion of mercury-resistant colonies at each timepoint. There was no effect of pQBR57 par overexpression on the emergence of segregants (p > 0.05 for all timepoints).*

pQBR57 has three ParB-domain proteins, PQBR57_0051, PQBR57_0055, PQBR57_0316. Only PQBR57_0055 has an associated gene predicted to encode a ParA-like protein (PQBR57_0054). PQBR57_0055 and PQBR57_0054 are syntenic and homologous (though with low sequence identity) to the pQBR103 *par* genes identified by Tett and colleagues (2007). We therefore believe that PQBR57_0054-0055 are the pQBR57 *par* system. However as we mention in the text, the activity of *par* systems is known to have side effects on gene expression, and we believe that it is these side effects

</td></tr>
</table>

	that we are witnessing here. We now add a few additional words on this subject (lines 640ff).
L. 156 and further: What does it mean to measure the fitness of the plasmids? Of course competition experiments that allow to calculate the Malthusian fitness of a strain relative to another are not to measure the fitness of the 'plasmids'? Do you mean fitness cost on the host? The traditional method measures the fitness of a plasmid-containing host relative to the plasmid-free host. The verbiage as it stands requires more context and explanation.	We apologise for the potential confusion here. During the course of an experiment, initially plasmid-bearing strains can lose the plasmid by segregation, or plasmid-free strains can gain it by conjugation. In this study, we calculate the fitness of initially plasmid-bearing 'test' strains relative to initially plasmid-free 'reference' strains (W = $\ln(\text{test}_{end}/\text{test}_{start})/\ln(\text{reference}_{end}/\text{reference}_{start})$). This approach tells us whether it is beneficial or costly to have a plasmid, and given this is the subject of the manuscript, is therefore the preferred method in this study. However, it is also possible to calculate the fitness of the plasmid, analogous to an allele spreading in a population, i.e. $W_{plasmid} = \ln(\text{plasmid-bearing}_{end}/\text{plasmid-bearing}_{start})/\ln(\text{plasmid-free}_{end}/\text{plasmid-free}_{start})$, where plasmid-bearing$_{end}$ includes transconjugants, and does not include segregants, and vice-versa for plasmid-free$_{end}$. This second approach tells us whether a plasmid is likely to spread in a population or not, and is what we mean by 'plasmid fitness'. We now define it more clearly (lines 157ff.).
From lines 159 - 163 we are to understand plasmid transfer can account for up to 10% of the estimated plasmid population, and measurements of 'plasmid fitness' (?) can increase fitness by 7%...shouldn't we be quite concerned about the effect of these numbers on competition results?	As described in our response above, these two calculated values describe different things. The subject of this study is the fitness costs of carrying a plasmid, and therefore we focus on tracking the fates of bacteria that initially carry the plasmid. If, instead, we track the fate of the plasmid (i.e. $W_{plasmid}$), this value is greater, because of the contribution of conjugation to plasmid success. However, $W_{plasmid}$ is not as useful a measure for understanding fitness costs because changes to $W_{plasmid}$ might be due to changes in conjugation rate. The discrepancy between the calculated values thus reflects the underlying biology, which we investigate directly (by measuring conjugation rate). We do not believe they cause concern for our results or interpretation. We hope that our editing of this section better explains our motivations and improves clarity.
Also related: Figure 2 (B) has Y-axis of 'corrected' relative fitness… What does this mean? the way I understand it, you are tracking the two competitors based on their chromosomal mutations and you are ignoring that the plasmid may have transferred to the competitor or may have been lost?	Figure 2B presents data from experiments where plasmid-bearing strains were competed against plasmid-free strains. In contrast to Figure 2A, the markers used to distinguish 'test' and 'reference' competitors were switched for some of the measurements in Figure 2B. To accommodate potential marker effects, fitness values were corrected by dividing each measurement by the marker effect, calculated as the mean fitness of plasmid-free strains. This is described in line 239ff.
Loss seem negligible (but was it tested for both plasmids and also when complementing cloned genes?).	Yes, we tested for both plasmids when performing fitness assays, and for pQBR57 when complementing cloned genes. We have also specifically looked into segregational loss in the presence of pQBR57_par, as described above (Supplemental Figure S8). We therefore do not believe that segregational loss makes an important contribution to the findings of our study.
A high (10%) transfer frequency would slow down the originally plasmid-free competitor with a costly plasmid. Thus in the case of competition between plasmid-bearing (P+) and plasmid-free (P-) strains, the cost of the plasmid would be underestimated (overestimating the relative fitness of P+)	We agree that, in principle, 'spiteful' infection of competitors with costly plasmids could potentially increase the relative fitness of a focal strain, but we do not think that this dynamic has a significant effect on our findings here. If competitor infection played a big role, we would expect to see fitness (W) correlating with conjugation rate, with increased conjugation rate associated with higher fitness. However we do not see this: there is no main nor any interaction effect of conjugation rate on fitness. See **Figure R1.**

<table>
<tr>
<td></td>
<td>
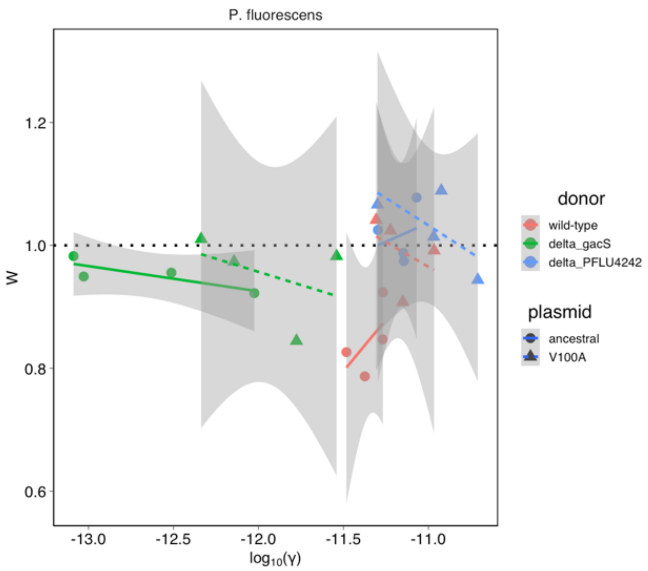

Figure R1. There is no correlation between plasmid fitness and conjugation rate. *Plot of conjugation rate against fitness, using data collected as part of the conjugation experiment presented in Figure 1C. Lines indicate linear model fit with shaded areas indicating standard error. There was no significant effect of conjugation rate on W_{test}, or any significant higher-order interactions between conjugation rate and donor or plasmid.*
</td>
</tr>
<tr>
<td>Line 553: "...the direct effect that GacA/S has on PFLU4242 transcription can effectively provide a unified explanation for the two chromosomal modes of amelioration, converging on PFLU4242." This could be tested directly and would strengthen a paper seeking molecular mechanisms. Use the ΔGacS strain(pQBR) and express PFLU4242 from a vector: if fitness decreases, then it really is the downregulation of PFLU4242 in ΔGacS strains that ameliorated cost, eliminating the confounding effect of the 700+ genes under GacS control.</td>
<td>
Following the Reviewer's suggestion, we tested this by inserting either empty pME6032, or pME6032 containing wild-type PFLU4242 cloned into the SacI/KpnI site into the ΔgacS mutant. We then introduced pQBR57 or pQBR103, and assessed growth rates with and without induction by IPTG. The results (below) show that PFLU4242 expression had no effect in the absence of the pQBR plasmids, consistent with our previous statement that PFLU4242 is not toxic by itself. However, introduction of either pQBR plasmid caused significant reduction in growth, consistent with our hypothesis that GacS exerts effects through PFLU4242. See lines 517ff and Figure S6.

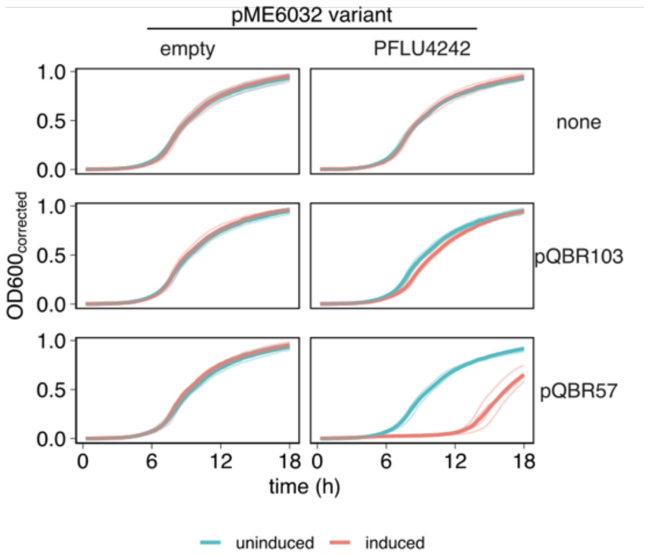

Figure S6. Overexpression of PFLU4242 recapitulates pQBR plasmid fitness cost in ΔgacS mutants. *Columns of panels indicate whether the expression vector was empty or contained PFLU4242. Each vector was transformed into P. fluorescens ΔgacS four times independently before conjugation with plasmid pQBR57 or pQBR103. Independent replicates are indicated with thin lines, and the mean across replicates is indicated by the thick line, for each induction condition. Comparison of the effect of induction on areas-*
</td>
</tr>
</table>

	under-the-curve showed that PFLU4242 expression had a significant effect in the presence of pQBR57 ($t_{5.04}$ *= 27, p < 0.001) or pQBR103 (*$t_{3.33}$ *= 5, p = 0.017) but not for pQBR-plasmid-free (*$t_{3.95}$ *= -0.05, p = 0.96).*
L. 810: "The principal group of genes upregulated by both plasmids were located in chromosomal regions annotated as mobile genetic elements." The authors should point out that potential conflict between a genomic island and a plasmid was (first?) pointed out by San Millan et al in P. aeruginosa and then Loftie-Eaton et al in Pseudomonas sp. The finding of potential conflict between chromosomally located and independently replicating mobile genetic elements in multiple species of Pseudomonas is intriguing.	We agree that this is an intriguing emerging pattern in plasmid-bacterial interactions. We now include these references in the discussion (lines 857ff.).
L. 604 and possibly other places in the manuscript: "Plasmid acquisition activates the SOS response,...". This may be misinterpreted as the event of acquiring the plasmid activating the SOS response, but it is not. As the authors explain elsewhere: the plasmid activates the SOS response long after it was acquired horizontally by conjugation, a sort of 'chronic' SOS response. It may be better to call this "plasmid carriage".	Thanks for this suggestion, this change improves the clarity of our argument here. We have also replaced 'acquisition' with 'carriage' in a few other parts of the manuscript, where appropriate.
It would be nice to show microscopically that individual cells with plasmid look different before amelioration - often cells are elongated due to SOS response, and cell lysis could be demonstrated with a live/dead staining.	We agree that single-cell level analysis of plasmid fitness costs and their consequences would be very interesting, but this goes beyond the scope of the current study.
L 26: plasmids don't have 'expression levels'. Maybe 'plasmid gene expression levels', or 'copy number'.	Thanks for the suggestion, we have made a change accordingly (line 25).
Line 28: 'This single chromosomal locus acts as a key mediator of plasmid fitness cost'… for pQBR-like plasmids.	We accept the Reviewer's point that plasmid-bacterial conflicts are likely to be gene and plasmid specific, but in this case we believe that the context makes this clear.
L. 60: how do you distinguish between bacterial growth and replication? Can you clarify?	Bacterial growth implies increase in biomass *per se*, whereas replication refers to increase in number of cells. These phenomena tend to be correlated, but can be distinguished on the basis of e.g. microscopy and dry weight measurements. However, we touch on this subject only very briefly in order to explain plasmid fitness costs. We believe that expanding on this would be tangential to the matter at hand and would lead to more confusion amongst readers. We have therefore edited to read "reproduction" rather than "replication, growth" (line 58)
L. 265; mechanisms OF plasmid compensation.	Thanks — this has been corrected (line 277).
Paragraph starting on L. 395: wasn't this already done by Harrison et al for one of these plasmids? More context would be helpful.	Our previous paper (Harrison et al. 2015) reported microarray data comparing plasmid-free SBW25 against ancestral and experimentally evolved SBW25(pQBR103). Here, we expand this analysis using higher resolution RNAseq to include (i) comparative analysis of the transcriptional impact of multiple distinct plasmids, and (ii) comparative analysis of the transcriptional effects of multiple, distinct, defined compensatory mutations that we engineered into the ancestral SBW25 background. We therefore believe that the new data is a major advance on the previously published work.
L 399: 'GO terms' is not defined?	Thanks — we now define (line 397).
Fig. 4, L. 490: "red indicates..." comes right after you explain the "coloured symbols" below each gene, yet now red/blue refer to differential expression levels, this could confuse the reader; swap sentences?	We have made this change in the legend for Figure 3 and Figure 5.
L. 521: "causing translation": do the authors mean "allowing translation to occur"? Basically	We have clarified this sentence (line 505).

Rsm is not or less active, and thus does not inhibit translation (or less so)? And translation of what?	
L. 559: difficult sentence; try to rephrase.	We have rephrased (line 538ff).
Fig. 6 and other relative fitness figures: it is not always clear against which strain these strains were competed against. The legend says "Relative fitness' but it doesn't say relative to what. In the Methods (L 150) the authors clearly state "For all competitions, GmR strains were competed against SmR-lacZ [43] strains.", but it may be helpful to remind the reader of that in the figure legends. Is it always the plasmid-free SmR-lacZ or sometimes the plasmid-containing?	All our plots show fitness of plasmid-containing relative to plasmid-free strains. In most cases the plasmid-free strain is the SmR strain, but in some cases (Figure 2B) the plasmid-free strain is the GmR strain. We explain this more clearly in the Methods (line 150ff.) and explain that the reference strain for all competitions is the plasmid-free strain in the legend to relevant figures.
I am a bit lost with Fig. 6: Was the strain with mutated plasmid (and with no insert or vector) indeed more fit than the same strain without plasmid? And the cloned mutated plasmid gene does not completely restore the fitness cost. This paragraph describing Fig. 6 needs to be more clear.	The key comparison here is between x-axis 'ancestral' and 'no insert', and how this is affected across the panels: crucially, a cost of the 'ancestral' insert appears when the V100A variant of pQBR57 is present. We also cloned the V100A mutant as a control; if our hypothesis was correct then the V100A mutant would have no impact on the fitness of either ancestral or V100A variant pQBR57. This was largely the case, though we did see a small negative effect in the presence of the ancestral pQBR57, probably because, when expressed at high levels, there was some residual activity of PQBR57_0059_V100A. This is now discussed more fully in that paragraph (line 615ff.).

Reviewer 3

**Suggestion**	**Response**
From Fig 2A and S1B it is not clear to me if gacS deletion fully compensates the cost of pQBR57, could you specify the statistical results?	The *gacS* mutation results in fitness measurements for pQBR57-carrying bacteria that appear intermediate between uncompensated wild-type bacteria carrying pQBR57, and fully-compensated (ΔPFLU4242 with pQBR57), or plasmid-free bacteria, and so we do not believe that *ΔgacS* fully compensates pQBR57. The statistics are ambiguous: *ΔgacS*(pQBR57) are not significantly different from wt(pQBR57), nor from wt(plasmid-free). We now specify this in the text (lines 311ff)
In Fig 1C (for intraspecific conjugation) there seems to be a small effect from the interaction between the gacS deletion and PQBR57_0059 variants. Do the two PQBR57_0059 variants differ significantly in the gacS mutant?	No, the difference here is not statistically significant, though it is approaching the margin ($t_{43} = 2.99$, $p = 0.055$).
The statement in line 772 is only valid for pQBR57, correct? How do you reconcile the PFLU4242 mediated fitness cost in the pQBR103 carrying strain, given that this plasmid already downregulates PFLU4242 to the same level as the gacS deletion (lines 535 and 781)?	We show in Figure S5 that PFLU4242 expression is increased in wild-type and pQBR57 strains when *gacS* is knocked out. We now also show in Figure S6 that overexpression of PFLU4242 in a ΔgacS pQBR57 strain is sufficient to recapitulate a fitness cost. The situation with pQBR103 is more complex, since pQBR103 appears to have an effect on PFLU4242 that is independent of gacS. However, there is a significant effect of PFLU4242 expression in the ΔgacS pQBR103 strain (Fig. S6), which, though less extreme than for pQBR57, shows that PFLU4242 acts as a proximal cause of fitness costs for both plasmids.
Could this be the reason why the cloned par locus has no effect on pQBR103 amelioration? Interestingly, the par genes from pQBR103 also seem to be upregulated (less than 2-fold?) in the gacS mutant (fig S4 & S6). Would the native par system of pQBR103, instead of that from pQBR57, be able to ameliorate the cost of pQBR103 (line 692)?	This is a very interesting question, and something we do speculate on briefly (lines 807ff). Par systems could pose interesting questions for plasmid fitness costs since they are common on low-copy conjugative plasmids, but (as our data suggests) their effects on fitness are likely to be plasmid-specific. We are looking forwards to testing these questions, as well as unpicking the underlying the molecular biology, in future work.
PFLU4242 disruption leads to upregulation of pQBR103 tra and pil genes. Could this explain why the conjugation rate of this plasmid did not increase (line 382)?	This is an interesting hypothesis. The presence of the Type IV pilus genes (pil) on both pQBR57 and pQBR103 raises intriguing questions about their functions — whether they are involved in conjugation, or some other aspect of bacterial activity. However, we are hesitant to speculate too much on this given the lack of hard data and length of the paper as it stands.
Do the plasmids originally carry copies of Tn6291, or could they have acquired it during the experiment? Could transposition and consequently Tn6291 increased copy number explain the upregulation of Tn6291 genes (line 497)?	The plasmids do not carry copies of Tn6291. We showed in our previous study that pQBR57 acquired Tn6291 during an long-term evolution experiment (Hall et al. 2017 doi: 10.1038/s41559-017-0250-3) but this is not the case for the short term culture that we extracted RNA from. We know this because, of the 24 genes on the transposon, only the three transposase-associated genes were upregulated in plasmid-containing strains. Additionally, as each replicate was from a different conjugation event, it is very unlikely that transposition would have randomly occurred in all plasmid-bearing samples but not in the plasmid-free samples. See Figure R2.

	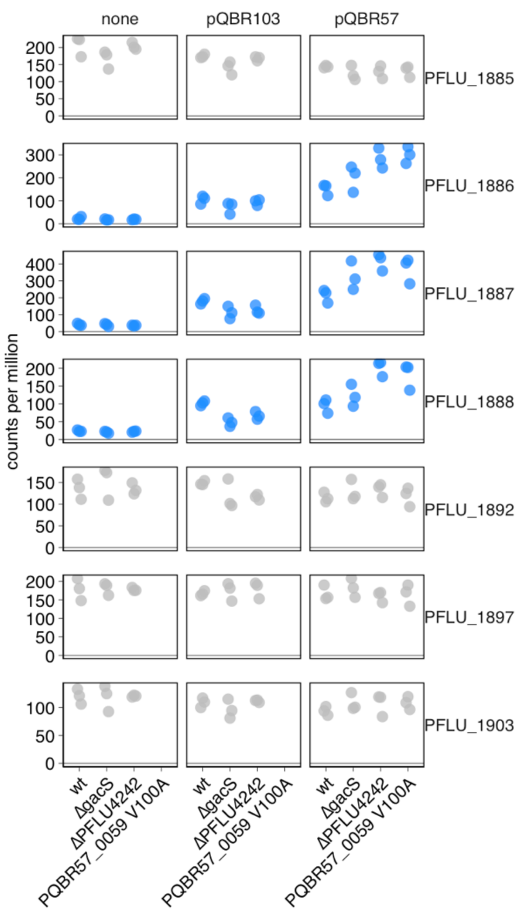<br>***Figure R2. Only the transposase genes of Tn6291 are upregulated.*** *Raw counts-per-million values for selected genes on the Tn6291 transposon under different conditions. Points indicate data from individual biological replicates. The three transposase-associated genes are in blue, in grey are a selection of representative Tn6291 genes. Panels are arranged with plasmid treatments in columns, genes on rows, and ameliorations on the x-axes.*
The statement in line 705 could be supported by the work of Hideaki Nojiri with pCAR1, specifically PMID: 17675379, where it is shown that chromosomal transcriptional changes are due to specific cross talk between plasmid and chromosomal encoded par genes. In that work, the one cited in reference 71 and PMID: 24889869, it is also shown that pCAR1 upregulates the expression of prophage genes. I think a discussion of these papers would enrich this manuscript.	We agree that our discussion would be improved by more discussion in relation to the work of Hideaki Nojiri's lab, and we now add a couple of additional sentences on this subject (lines 744, 821, 837, 862ff.).
The statement in line 707, "furthermore describes the molecular negotiation by which this occurs", may be too bold as this is not a full description since there are still some unknowns in the mechanism provided in Fig. 8.<br>The authors show in this work that plasmid costs are due to specific genetic conflicts, in this case SOS induction that consequently induces prophage genes. Would it be relevant to discuss the interplay between plasmids and accessory elements (single genes or mobile genetic elements such as phages) as a source of cost, rather than the plasmid-host interaction? Indeed, the fitness costs of other plasmids have been shown to derive from conflicts with horizontally transferred helicase genes (references 25 and 32).	We take the reviewer's point and tone this sentence down (line 702). We also include a new paragraph discussing the exciting theme emerging from similar studies (line 857ff).

In lines 23, 88-92, 363 and 774, the authors suggest that costs arising from general plasmid properties, such as the requirement of the replication and transcription/translation machineries, would require multiple or large (deletions) amelioration events, while specific gene interactions could be resolved by more targeted solutions. I hope I did not misunderstand the authors point of view. Assuming I understood it correctly, I only agree partially, due to the global effects of two component systems and other general transcription regulators. In the present work, for example, targeted solutions for the specific conflict exist such as the disruption of PFLU4242 and PQBR57_0059, but so does the disruption of gacAS. The latter, by regulating a variety of genes, could compensate simultaneously more general costs. Indeed, the initial interpretation of the results from the work in reference 33, could be that the proximal cause of plasmid cost was translational demand. The current manuscript however shows that the conflict is more specific. The point being that disruption of global regulators provides a confounding factor in what concerns the identification of the proximal causes of fitness costs, since both specific or general conflicts can be targeted. Therefore, this kind of interpretation is not trivial and the discussion of this matter should be more careful.	We now address the interaction between the two-component regulator GacA/S and PFLU4242 in causing plasmid fitness costs directly in a new experiment presented in Figure S6. We show that when a single gene from the GacA/S regulon — namely PFLU4242 — is expressed from an inducible vector in a ΔgacS mutant, the fitness costs of pQBR57 and pQBR103 return. We believe that these results (reproduced in the response to Reviewer 2 above) help to tease apart the general and specific effects of gacS, and suggest that the global effects of GacA/S on gene expression have probably only a small effect on plasmid fitness costs. See lines 521ff and Figure S6.
Lines 160 - 163: could you elaborate on this? I do not understand the message the authors try to convey with this information.	We include more information in response to a similar question fromReviewer 2 and have rewritten and expanded on these lines to increase comprehensibility.
Was conjugation performed in solid or liquid media (line 132)? I also did not see a methods section concerning the conjugation reported in line 367.	Conjugations were performed in liquid KB, we specify this more clearly now (lines 130ff.).
In Fig S2, 12 pQBR57 variants are shown, 5 with an intact PQBR57_0059, and 7 with variants of this gene, but 10 variants are mentioned in line 334. Moreover, could clarify in the legend of Fig S2 what are the loci affected in variants A01, A11, C01, C10 and C11? The locus_tags alone are not very information, are all these encoding hypothetical proteins?	Yes, this is correct — we describe two of the variants in detail, A01 ('anc') and B09 ('V100A'), and then explain that 10 other (additional) variants were tested. However I can see how this might have been unclear, and so have re-phrased. I also realized that I neglected to indicate 'anc' and 'V100A' in Figure S2; these variants are now labelled.<br><br>As described above the plot and in the text, variant A01 had no mutations. The other variants mentioned by the Reviewer had transposon insertions in various locations, with the locus tags of the affected regions provided above each column of sub-plots. Unfortunately there is little information about these predicted genes: all are hypothetical proteins. We now explain this in the caption.
PFLU5278 (shown in Fig. 5) is not labeled in Fig 3.	Correct — PFLU5278 did not qualify for inclusion in Figure 3 as it was not >2x upregulated with p<0.05.
There are two Figure 4 (lines 448 and 486) and correct the following references to figures: Fig. 2B (line 329), Fig. 5A. (line 644), Fig. 3 (line 419).	Corrected — thank you for your close attention, these changes were missed when we reordered the figures in an earlier draft.
The reference in line 1239 is missing from the reference list in the supplementary information. Please refer to Fig. S2 in the section starting in line 1266.	Corrected, thanks.
"reverse genetics" should simply be "genetics" (lines 27 and 115). Please be more precise genetic notation: "cloned as a fragment into the EcoRI/KpnI site of pUCP18" (lines 139 and 145), "SmR lacZ+ strains" (line 150), and "par locus" or "parAB genes" (lines 235, 239 and 665).	Corrected, thanks.

Line 55: Entry exclusion systems are features of other genetic elements, not bacterial host features.	This is true, and on reflection probably applies to restriction systems, CRISPR, and barriers to plasmid replication and segregation too. However, the issue becomes muddied when resident genetic elements become domesticated, and the boundary between an individual MGE and its host becomes blurred. This is an interesting point but somewhat tangential to that which we are trying to make. We therefore have made the first sentence less specific to bacterial 'hosts' and refer instead to 'recipient bacteria' (line 49).
Vogwill and MacLean's (PMID: 25861386) meta-analysis suggests that plasmid size is not correlated with fitness cost. This could be discussed in line 75.	This is a great paper, and we now refer to its findings in the introduction (line 91ff.).
The statement in line 102 could be supported by reference 28 (Porse et al. 2016), while the conclusion in line 402 could be supported by reference 26 (San Millan et al. 2018).	The Porse et al. 2016 reference seems to make a slightly different point to that which we are making, namely, while we are saying that the mechanism of plasmid cost defines what mutations are available for compensation, Porse and colleagues show that a particular beneficial compensatory mutation did not arise in one species (due perhaps to differential recombinogenic activity of IS26). We therefore refrain from adding this reference and have rephrased this sentence slightly to explain our point more clearly. The San Millan reference is appropriate and we include it as suggested.
It would be helpful to state plasmid relatedness early in line 256, as done in line 853.	We have now added a few words to the end of the paragraph on this subject.
Line 604 can be misinterpreted as plasmid acquisition immediately after conjugation. Replacing acquisition by carriage may avoid confusion.	Thanks — we have made this change.
A few typos: "orbital" (line 171), "acquisition" (line 395), "expression" (line 513), close the parenthesis (line 374).	Done — thanks for the close reading.

Reviewer 4

Suggestion	Response
The authors claim in the abstract and introduction (and discussion line 808) that they conduct 'experimental evolution', yet I couldn't find any evolution experiments (only competition over time?) but rather only citations of previous studies. Using previous results is not wrong and even a good practice in my opinion, but should definitely not be hidden from the reader that is given a false impression of the study.	Referring back to our previous experimental evolution studies enables us to amass compelling evidence for the generality and reproducibility of our findings, and we believe that this presentation helps to contextualize and strengthen the current work.<br><br>We have made changes to the Abstract to make this clearer (line 25), and added the appropriate citation to the sentence in the Discussion (line 845)
Furthermore, reading the previous study by Hall et al (DOI: 10.1099/mic.0.000862), reveals the discovery of the mutations in PFLU4242 and its biological description (fitness impact). Thus, taking both points together I remain concerned about the novelty of parts of the here presented results.<br><br>The authors should consider restructuring their manuscript to have a larger focus on the indeed very striking (and very exciting) transcriptomic analysis. One suggestion could be to start with transcriptomics (as the mutations are already published material) and have more emphasise on the mutated genes (maybe by phylogenetic analysis) and the ectopic cloning of the by transcriptomics identified candidate genes.	Previous work by our lab indeed identified PFLU4242 as a key target for compensatory mutations. The current work greatly extends our published studies, integrating experimental evolution results with a series of new experiments including competitive fitness assays, reverse genetics, and transcriptomics, that provide new insight into the molecular mechanisms underlying plasmid fitness costs and their resolution.<br><br>We are reluctant to restructure the manuscript as we consider the current structure — from identification of parallel mutations, to fitness impacts, to underlying mechanism — to be more intuitive for a general audience.<br><br>We note that though the mutations themselves are published, they have not been previously presented in this integrated way (Figure 1), which we believe adds valuable insight. We also note that while we have published some experiments on the fitness impacts of *ΔgacS* and *ΔPFLU4242*, the data presented in Figure 2A are new data that reproduce and strengthen these previous findings. Figure 2A also provides useful context for the other panels in the Figure, which address questions that have not been covered in previous work. For example, this is the first time that we have shown plasmid-borne compensatory evolution via mutation to PQBR57_0059, and the first time we have shown how different modes of compensatory evolution affect conjugation rate within and between species.
1) For the beginning of the manuscript, the authors should consider a deeper analysis of the genes in which compensatory mutations appear. For example, for PFLU4242, which is indeed an intriguing candidate. Here, I am missing a deeper description of the PFLU4242 gene itself. What predicted domains does it have (hhpred?)? I see some annotation in figure one, but am missing explanation in the text (or better representation of the domains).<br><br>In addition, does this gene have homologues in other species and. In general, it is not fully clear whether PFLU4242 is a housekeeping gene or rather may have accessory function. A deeper analysis in pseudomonas could help to understand the role of the gene.	We apologise for this oversight. We now include details about the domains of PFLU4242 (line 755ff).<br><br>We are confident that PFLU4242 is not a housekeeping gene, given the results of fitness assays and transcriptomic analyses presented in this manuscript. Additionally, as homologues of PFLU4242 are not found in all Pseudomonas, but are found in distantly-related species (Hall et al. 2019), PFLU4242 is clearly part of the 'accessory genome'. We now include a few lines both the structure of PFLU4242, and its distribution, in the discussion (line 755ff.)<br><br>Like the Reviewer, we are intrigued about PFLU4242, and are currently conducting an in-depth functional, structural, and bioinformatic study of PFLU4242, its homologues in other species, and its interactions with other plasmids. However, this is still in the preliminary stages, and is beyond the scope of the current study.
The same relates to the plasmid gene PQBR57_0059? Does is have homologs in other plasmids or phages? The authors should aim to draw a larger picture of the meaning of the genes and mutations here.	PQBR57_0059 is a member of the family of lambda-like transcriptional regulators, and diverse similar genes are found in other chromosomes, plasmids, and phage. A full evolutionary analysis of lambda repressor-like genes is beyond the scope of the current work, but we add a few words on this subject (lines 585ff).
2) In previous publications (e.g. Harrison et al.,2015) mutations in gacA/S had a strong	Harrison et al. (2015) found that gacA/S mutations affect various phenotypes, but competitive fitness in KB media was

negative impact on the host cell. How do the other interpret that they don't observe that in the current study? Such result would fit the observed changes in the transcriptome. In addition, the authors could speculate that mutations gacS/A may be not sustainable and other mutations (like in PFLU4242) might be more 'feasible' for the cells. The authors should include this in their discussion.	not greatly affected (Figure 3B from that paper). This is consistent with our findings here. The effect of gacA/S mutation is likely to emerge in more complex environments, where gacA/S regulated genes are adaptive. We have added a few more words on this subject to the Discussion (lines 770ff).
3) The persistence of the different plasmid mutations (Fig.S2). Many points are unclear in this figure. 1)Where are these mutations coming from? And what does 'naturally emerging' (line 334) mean? 2) Maybe I missed this information, but are these additive mutations? 3) The coloring is not clear, are there segregants (black) anywhere in the figure? The authors need to clarify the results of this figure in the text and the figure itself.	1. The mutations emerged in the evolution experiment reported in Hall et al. (2017, 2018), and were conjugated back into an ancestral recipient strain for these experiments. This is explained in line 119ff.<br><br>2. These mutations emerged during the evolution experiment. We have not been able to test directly whether they are additive or not. The purpose of this experiment was to test whether our findings were specific to the V100A mutation that we use in subsequent experiments, and the results show that different disruptions to PQBR57_0059 have a similar effect.<br><br>3. Very few segregants were detected in this experiment; we now explain this in the caption.
4) There is some mistake in the numbering of the figures (2x figure 4). The Figure 4(2) is not clear and needs to be modified.	We have corrected the figure numbering, thanks for noticing this.<br><br>We believe the (now correctly labelled) Figure 5 is clear. Please can the Reviewer provide further explanation about what requires modification?
5) Maybe I overlooked it, but in Fig. 5, the authors write corrected OD600. I couldn't find anything in the methods section. What did the authors correct for? This needs to be clarified.	OD600 was corrected by subtracting the absorbance of blank (no bacteria added) wells. We now explain this (lines 183-185).
6) Regarding figure 8 - How does the growth curve look of strains carrying the large plasmids that activate the tailocin? Is it comparable in regards to toxicity/ fitness decrease? In addition, should lysis not be visible in form of plaques on solid media? The authors should clarify.	The fitness effects of artificial PFLU1169 overexpression exceed those of pQBR plasmid carriage — this is to be expected as the P_{tac} promoter of the pME6032 expression vector drives a very high level of transcription. The purpose of these experiments is not to simulate exactly the transcriptional effects of pQBR plasmid carriage, but to understand the functional effects of the genes that are induced, and therefore direct growth curve comparisons with the plasmids are not likely to be enlightening.<br><br>Plaques are formed by lytic phage in a cycle of infection, lysis, and infection of neighbouring cells. We do not see this pattern with the SBW25 prophage 1, first, because prophage 1 is not an intact lytic phage, rather it acts as a toxin that does not package its own genetic material for self-replication, and second because, as with prophage, bacteria that carry tailocin genes are resistant to that particular tailocin (Carim et al. 2021).
1)How similar are the presented plasmids? It is important to state that these are (I assume) different rep types and in general plasmids of different origin.	We now provide some details about the presented plasmids (lines 280ff), and a reference for further information.
2) Figure 1 is very small (and not very intuitive), especially the writing.	We have increased the size of Figure 1, and we add more details about the domain text in the legend.
3) Did the authors ever attempt to study the effect of double (or consecutive) mutations? It might be not in the framework of this study, but perhaps the authors have data for it.	Yes, we look at double mutations in Figure S1.
4)There is some mistake in the numbering of the figures (2x figure 4)	This has been corrected.
