# Supplementary material for: Plasmid fitness costs are caused by specific genetic conflicts enabling resolution by compensatory mutation
Source: PLoS Biol. 2021 Oct 13;19(10):e3001225. doi: 10.1371/journal.pbio.3001225 (PMC8544851; doi:10.1371/journal.pbio.3001225)
Supplement: Attachment — Submitted filename: COMPMUT_REV_Response.pdf [file pbio.3001225.s011.pdf]

Editor Requests

a) Please address the remaining requests from reviewer #3.

Done, please see below for details.

b) Please provide a more informative title; maybe "Plasmid fitness costs are caused by specific genetic conflicts that can be easily compensated" or Plasmid fitness costs are caused by specific genetic conflicts rather than intrinsic plasmid properties"

We have retitled our manuscript "*Plasmid fitness costs are caused by specific genetic conflicts enabling resolution by compensatory mutation*".

c) Many thanks for providing the data and code needed to reconstruct the Figures in Github. Please could you cite this location for the data clearly in each relevant main and supplementary Figure legend? e.g. "The data underlying this Figure may be found in https://github.com/jpjh/COMPMUT/" (we suggest using this rather than the institutional repository which is likely to be less robust in the long term)

Done. If requested I'm happy to upload the data files as supplementary information to PLOS as supplementary information as well.

As you address these items, please take this last chance to review your reference list to ensure that it is complete and correct. If you have cited papers that have been retracted, please include the rationale for doing so in the manuscript text, or remove these references and replace them with relevant current references. Any changes to the reference list should be mentioned in the cover letter that accompanies your revised manuscript.

I have checked the reference list. As far as I am aware, no cited papers have been retracted.

DATA NOT SHOWN?

- Please note that per journal policy, we do not allow the mention of "data not shown", "personal communication", "manuscript in preparation" or other references to data that is not publicly available or contained within this manuscript. Please either remove mention of these data or provide figures presenting the results and the data underlying the figure(s).

I have repeated the analyses that this referred to and now add a link to the output on the Github and the DataCat repository. We have revised the manuscript text accordingly.

Reviewer 3 Requests

I believe the G force values should be 1000-fold higher in the Methods Section "RNA extraction and sequencing".

This has been corrected, thanks.

Fig 4B mentioned in line 646 should be Fig 5B, while Fig 8B mentioned in line 666 should be Fig 8A.

Thanks — this has been corrected, along with another couple of figure references were missed in the updates.
